# Effects of the Ayurved Siriraj Wattana recipe on functional and phenotypic characterization of cytokine-induced killer cells and dendritic cells in vitro

**DOI:** 10.1186/s12906-016-1480-7

**Published:** 2016-11-29

**Authors:** Adisak Wongkajornsilp, Nuntarak Numchaisermsuk, Khanit Sa-ngiamsuntorn, Pravit Akarasereenont, Valla Wamanuttajinda, Kanda Kasetsinsombat, Sunisa Duangsa-ard, Tawee Laohapan, Kittipong Maneechotesuwan

**Affiliations:** 1 Department of Pharmacology, Faculty of Medicine Siriraj Hospital, Mahidol University, Bangkok, 10700 Thailand; 2 Center of Applied Thai Traditional Medicine, Faculty of Medicine Siriraj Hospital, Mahidol University, Bangkok, 10700 Thailand; 3 Department of Medicine, Faculty of Medicine Siriraj Hospital, Mahidol University, 2 Wanglang Road, Bangkoknoi, Bangkok 10700 Thailand; 4 Department of Biochemistry, Faculty of Pharmacy, Mahidol University, Bangkok, 10400 Thailand

**Keywords:** CIK cells, Dendritic cells, Lymphocytes, Traditional medicine Asia, Immunomodulation, Cytotoxicity

## Abstract

**Background:**

Ayurved Siriraj Wattana recipe (AVS073), has been prescribed as tonic, to increase appetite, and for pain relief. It also exhibits antioxidant, anti-inflammatory, immunomodulating and anti-cancer activities. However, the immunomodulatory effects on antigen-presenting cells and effector T cells remained elusive. We thus aimed to study the effects of AVS073 on differentiation, maturation, functions and proportions of CIK cells and monocyte-derived DCs.

**Methods:**

CIK cells and monocyte-derived DCs were treated with AVS073, followed by the assessment of T-helper (Th) phenotypes using real-time RT-PCR and flow cytometry.

**Results:**

AVS073 promoted Th1 phenotype in CD3^+^CD56^+^ subset of CIK cells through increasing STAT4, T-bet, and interferon-γ. AVS073 inhibited Th2 phenotype through decreasing STAT6. AVS073 inhibited Treg phenotype through decreasing STAT5A, STAT5B and IDO. AVS073 promoted Th17 phenotype through increasing STAT3, RORC and IL-17. AVS073 treatment of mDCs resulted in increasing Th1-prone cytokine (IL-12) and Th17-prone cytokines (IL-6 and IL-23).

**Conclusions:**

AVS073 upregulated Th1 and Th17, but downregulated Th2 and Treg phenotypes within CD3^+^CD56^+^ cells. The treatment of mDCs drove Th1 and Th17-polarizations.

**Electronic supplementary material:**

The online version of this article (doi:10.1186/s12906-016-1480-7) contains supplementary material, which is available to authorized users.

## Background

Ayurved Siriraj Wattana recipe (AVS073) [1] has been used in Thai traditional medicine for decades. The recipe was prescribed for health promotion, strength supplement, appetite induction and attenuation of degeneration (anti-aging). It has recently been tested for immunomodulatory activity (NK cells activity), osteoarthritis and gastric emptying rate [2–4]. It conveyed protection against UVA-induced melanogenesis through an antioxidant/redox mechanism [1]. The 18 medicinal plant components of the recipe are *Aegle marmelos* (L.) Corrêa., *Boesenbergia rotunda* (L.) Mansf., *Caesalpinia sappan* L., *Carthamus tinctorius* L., *Cinnamomum siamense* Craib, *Citrus sinensis* L.Osbeck, *Cladogynos orientalis* Zipp. ex Span., *Cryptolepis buchanani* Roem. & Schult., Cyperus rotundus L., *Derris scandens* (Roxb.) Benth., *Drypetes roxburghii* Wall., *Ferula assa-foetida* L., *Ligusticum sinense* Oliv., *Mallotus repandus* (Willd.) Müll.Arg., *Piper nigrum* L., *Saussurea lappa* (Decne.) Sch.Bip., *Terminalia chebula* Retz., *Tinospora crispa* (L.) Hook. f. & Thomson.

*Aegle marmelos* (L.) Corrêa has been used in the treatment of chronic diarrhea, peptic ulcers and dysentery, as a laxative and to recuperate from respiratory affections in various traditional medicines [5, 6]. *Boesenbergia rotunda* (L.) Mansf has been commonly used in Southeast Asia as a food ingredient. Traditional healers throughout Thailand have been using this plant against inflammation, aphthous ulcer, dry mouth, stomach discomfort, dysentery, oral disease and cancers [7–9]. The heartwood of *Caesalpinia sappan* L. has been used as a hemostatic, analgesic and anti-inflammatory for traumatic disease and blood flow promoting agent [10–13]. *Carthamus tinctorius* L. has various pharmacological effects, e.g., anticoagulant and antithrombotic activities, anti-fibrotic effect, immunomodulatory activity [14–16]. The stem of *Cryptolepis buchanani* Roem. & Schult. has been used for the treatment of inflammation, including muscle and joint pain [17–19]. *Cyperus rotundus* L. has antidepressant [20] and anti-melanogenesis activities [21]. *Tinospora crispa* (L.) Hook. f. & Thomson has immunostimulatory effect [22, 23]. The other plant components in AVS073 also have anti-inflammatory effect and analgesic activity [24–30]. Some components of AVS073 showed direct anti-cancer properties through inhibiting cell growth or inducing cellular apoptosis, and indirect pathway via the immunological action of immune cells [31, 32]. Several in vitro and in vivo studies demonstrated that the extracts of AVS073′s components carried antioxidant, anti-inflammatory, immunomodulating and anti-cancer actions [33–40].

Differentiation of Th1 cells are driven by STAT1 and STAT4 while STAT6 and GATA3 induces Th2 cells, forkhead transcription factor (Foxp3) induces regulatory T (Treg) cells, and retinoic acid-related orphan receptor (Rorc) induces Th17 cells [41]. Differentiation of Th1 cell and regulatory T (Treg) cells may be actually linked to the differentiation of Th2 and Th17, respectively, depending on the overall cytokine milieu. The differentiation of both Treg and Th17 cells requires TGF-β. The differentiation of Th17 cells requires low concentrations of TGF-β in combination with the pro-inflammatory cytokines IL-6 and IL-23 [42], while in the absence of pro-inflammatory cytokines, high concentrations of TGF-β is optimal for Foxp3 expression and thus tips the balance towards Treg cell differentiation [43, 44]. In addition to cytokine environment, Treg cell development could be enhanced by kynurenine [45], a breakdown product of indoleamine 2, 3-dioxygenase (IDO) in dendritic cells (DCs).

DCs are professional antigen presenting cells that present antigens to naïve T cells, inducing their differentiation towards either Th1 or Th2 phenotype. DCs that generate Th1 responses would handle infections or malignant disorders via the induction of Th1-polarizing cytokine interleukin-12 (IL-12) and interferon-gamma (IFN-γ) [31]. In addition, Th17 cells also play a role in anti-tumor immunity [46]. In contrast, induction of Th2 responses by DCs may provide clinical benefits when Th1 responses are excessive, e.g., transplantation, contract allergy, or autoimmune disorders, by producing Th2 cytokines in particular IL-4, to induce B cells to secrete protective antibodies [47]. Immature DCs can be differentiated from monocytes and bone marrow progenitor cells by treatment with granulocyte macrophage colony-stimulating factor (GM-CSF) and IL-4. Immature DCs are stimulated with maturation signals, such as tumor necrosis factor α (TNF-α), to express a strong immune response against foreign antigens. The exposure of cytokine-induced killer (CIK) cells to mature DCs led to the enhancement of anti-tumor cytolytic activity of the former [48–50].

A number of studies showed the possible immunological action of AVS073′s components in DCs. *Carthamus tinctorius* L. extract has been reported to stimulate the production of IFN-γ and IL-10 in mouse splenic T lymphocytes and promote the expression of maturation markers in mouse bone-marrow-derived DCs. Furthermore, *Carthamus tinctorius* L. treated DCs maintained the high profile of maturation markers in tumor antigen pulsed-DCs [31]. In contrast, *Piper nigrum* L. significantly inhibited the phenotypic maturation, cytokine production (TNF-α and IL-12), phosphorylation of ERK and JNK, but enhanced the endocytosis activity of LPS-induced bone-marrow-derived DCs [32]. DCs, which can subsequently interact with CIK cells [49, 51], might be potential targets of this recipe.

CIK cells have been used as non-major histocompatibility complex (MHC)-restricted effector cells with high cytotoxicity against a variety of tumor targets [52]. CIK could be driven toward Th1 phenotype away from Th2 phenotype, but no data for Treg and Th17 polarization [49]. However, there was no data available for the effect of AVS073 components on CIK cells. We investigated the alteration to Th-polarizing cytokine profiles in DCs and Th polarization in CIK cells after AVS073 treatment.

## Methods

### Preparation of Ayurved Siriraj Watana Recipe powder

The Ayurved Siriraj Watana Recipe was manufactured as powder under GMP Guidelines by the Center of Applied Thai Traditional Medicine, Faculty of Medicine Siriraj Hospital, Mahidol University. The sources of all herbal components came from the wild stretching from the Central and the Northeastern parts of Thailand. They were authenticated by experts, including certified pharmacognosists of the Center of applied Thai traditional medicine. Herbal raw materials were washed and dried in a hot air oven in accordance with the Ayurved Siriraj Watana Recipe Master Formula. It was validated with chemical fingerprint using ultra-performance liquid chromatography as previously described [1]. The crude powder was preserved at 25 °C in a desiccator. To prepare the extract, the crude powder was dissolved in 80% ethanol at a final concentration 100 mg/mL. The extract was filtered with cotton wool and subsequently centrifuged at 10,000 × g for 10 min. The supernatant was evaporated and lyophilized to dry powder.

### Generation of CIK cells and DCs from peripheral blood mononuclear cells

CIK cells and DCs were generated from peripheral blood mononuclear cells (PBMCs) of healthy donors and characterized as described previously [49–51]. All healthy donor volunteers understood and signed the informed consent document before the participation. The protocol was approved by the Institutional Review Board of the Faculty of Medicine Siriraj Hospital, Mahidol University. PBMCs were isolated from whole blood by Ficoll gradient centrifugation (IsoPrep®, Robbins Scientific, CA). The cell suspension was allowed to adhere over the container at a density of 5 × 10^6^ cells/mL for 1 h at 37 °C in RPMI 1640, 10% FBS, 100 U/mL penicillin, and 100 μg/mL streptomycin. The non-adherent cells (PBLs) were processed into CIK cells. The adherent cells were processed into mature DCs (mDCs) (Additional file 1).

To generate CIK cells, PBLs were maintained in RPMI 1640 (Invitrogen, Carlsbad, CA), 10% FBS (Biochrom, Berlin, Germany), 100 U/mL penicillin and 100 μg/mL streptomycin. IFN-γ (1000 U/mL, Amoytop Biotech, Xiamen, China) was added and incubated at 37 °C, 5% CO_2_ for 24 h. After 24-h incubation, 50 ng/mL monoclonal antibody against CD3 (eBioscience, CA), and 300 IU/mL IL-2 (Amoytop Biotech, Xiamen, China) were added. Recombinant IL-2 and fresh growth medium were added every 3 d. After 14 d, AVS073 were added to the culture for 7 d or left with medium alone.

The adherent PBMCs was maintained in growth medium without cytokine to be remain as monocytes. To generate immature DCs (iDCs), the adherent cells were cultured in RPMI 1640, 10% FBS, 400 U/mL GM-CSF and 500 U/mL IL-4 for 14 d. The differentiation into mDCs could be achieved by adding 1000 U/mL TNF-α (Amoytop Biotech, Xiamen, China) for 48 h. The mDCs were incubated with AVS073 for 7 d either before or after maturation induction with TNF-α to generate pre-treated mDCs or treated mDCs respectively.

### Preparation of CD3^+^CD56^+^ subsetof

An aliquot of 10^8^ cells of day 14 CIK cells were harvested. CD3^+^CD56^+^cells were isolated from CIK cells using CD3 microbead followed by CD56 Multisort Kit (Miltenyi Biotec, Germany) according to the manufacturer’s instruction. The CD3^+^CD56^+^ cell pellet was washed by adequate volume of the buffer, counted, and determined for viability by trypan blue exclusion. CD3^+^CD56^+^ cells were resuspended in appropriated volume of growth medium (RPMI-1640, 10% FBS, and 300 IU/mL IL-2) to achieve a density of less than 1 × 10^6^ cells/mL.

### Determination of cell proliferation

(3-(4,5-dimethylthiazol-2-yl)-2,5-diphenyl) tetrazolium bromide was dissolved in the culture medium (200 μg/mL). The solution was added to each well. The plates were incubated at 37 °C in 5% CO_2_ for 1 h. After which time, the medium was discarded and 100 μL of dimethyl sulfoxide was added. The plates were left at room temperature for 10 min with occasionally gently shaking. The absorbance in each well was measured at 595 nm and calculated as the percentage of the control as follows:

$$ \%\mathrm{proliferation}=\frac{\mathrm{OD}\left(\mathrm{sample}\right) - \mathrm{O}\mathrm{D}\left(\mathrm{background}\right)}{\mathrm{OD}\left(\mathrm{control}\right) - \mathrm{O}\mathrm{D}\left(\mathrm{background}\right)}\times 100 $$

OD (sample) represents the OD of the well containing the treated cells. OD (control) represents the OD of the well containing untreated cells. OD (background) represents the OD of the blank well.

### Fluorescence-activated cell sorting (FACS) analysis

PBLs were analyzed with fluorochrome-conjugated antibodies against CD3, CD8-, CD11a-, CD16-, CD28, CD56, CD69, CD152, CD154, CD278 (ICOS), CD279 (PD-1) (eBioscience, CA) and antibodies against regulatory T (Treg) cells (Biolegend). CIK phenotypes were analyzed with fluorochrome-conjugated antibodies against CD3, CD8, CD28, and CD56. DC phenotypes were analyzed with the following monoclonal markers: CD1a, CD11c, CD14, CD40, CD80, CD83, CD86 and HLA-DR (eBioscience). Cells were incubated with the corresponding fluorochrome-conjugated primary monoclonal antibodies at 4 °C for 30 min in the dark in 5 mL polystyrene round-bottom tube. The cells were washed by adding 2 mL of FACS buffer and pelleted by centrifugation at 400 × g at 4 °C for 5 min. The cells were resuspended in 200 μL FACS buffer. Regulatory T (Treg) cells were detected in PBLs and CIK cells by Human Treg Flow™ kit included Foxp3 Alexa Fluor® 488 and CD4 PE-Cy5/CD25 PE cocktail, according to the manufacturer’s protocol. Flow cytometry analysis on 30,000 cells was performed using a FACSCalibur (Becton Dickinson, CA). Data were analyzed using FlowJo version 10.0.7.

### RNA preparation and quantitative real-time PCR analysis

After AVS073 treatment, DCs or CD3^+^CD56^+^ cells were separately extracted for total mRNA by RNAspin mini RNA isolation kit (GE Healthcare, UK) according to the manufacturer’s instruction. Total mRNA was converted to cDNA using Improm-II™ reverse transcription system (Promega, Madison, WI). The cDNA samples were tested for quality and quantity by NanoVue™ Spectrophotometer (GE Healthcare, UK). The specific primers for DCs and CD3^+^CD56^+^ cells (Table 1) were designed by Vector NTI version 10 and Primer Express 3.0 (Applied Biosystems, CA) and purchased from 1^st^ BASE (Singapore). The specific genes were amplified with Brilliant® II SYBR® Green QPCR master mix (Agilent Technologies, Waldbronn, Germany) in a StepOnePlus real-time PCR system (Applied Biosystems). Quantitative real-time PCR was performed using 300 ng of cDNA in a 7.5 μL of SYBR master mix containing 20 μM primers and constituted to 15 μL with water and amplified for 40 cycles of 95 °C for 15 s, 56–60 °C for 40 s, and 72 °C for 40 s. The obtained Ct’s were subtracted with the Ct of GAPDH of the same condition to obtain ΔCt. The ΔCt’s of the treated cells were subtracted with ΔCt’s of the untreated cells of the same period to obtain ΔΔCt’s. The fold-changes could be obtained from the expression of 2^-ΔΔCt^.

**Table 1:** Primer pair and their information for quantitative real-time PCR

Genes	Oligonucleotides (5′ → 3′)	Size (bp)	Annealing (°C)
GAPDH	Forward: GAAATCCCATCACCATCTTCC	124	60
	Reverse: AAATGAGCCCCAGCCTTCTC		
IDO	Forward: AGTCCGTGAGTTTGTCCTTTCAA	68	60
	Reverse: TTTCACACAGGCGTCATAAGCT		
IFNγ	Forward: GTGTGGAGACCATCAAGGAAGAC	80	60
	Reverse: CAGCTTTTCGAAGTCATCTCGTTT		
IL-4	Forward: AACAGCCTCACAGAGCAGAAGAC	101	60
	Reverse: GCCCTGCAGAAGGTTTCCTT		
IL-6	Forward: GCTGCAGGCACAGAACCA	68	60
	Reverse: ACTCCTTAAAGCTGCGCAGAA		
IL-10	Forward: CTGGGTTGCCAAGCCTTGT	100	60
	Reverse: AGTTCACATGCGCCTTGATG		
IL-12	Forward: GCAAAACCCTGACCATCCAA	100	60
	Reverse: TGAAGCAGCAGGAGCGAAT		
IL-17	Forward: ACCTGTGTCACCCCGATTGT	90	58
	Reverse: GGGTCGGCTCTCCATAGTCTAA		
GATA3	Forward: ACTACGGAAACTCGGTCAGG	100	60
	Reverse: CAGGGTAGGGATCCATGAAG		
STAT1	Forward: GTGGCGGAACCCAGGAAT	97	60
	Reverse: TGACAGAAGAAAACTGCCAACTCA		
STAT3	Forward: ACCAAGCGAGGACTGAGCAT	90	58
	Reverse: TGTGATCTGACACCCTGAATAATTC		
STAT4	Forward: TTCCTTCTGTTTTTATCCCCATCT	128	60
	Reverse: TGTTGTGGGACTCAGGTTTTCTC		
STAT5A	Forward: CACGCAGGACACAGAGAATGA	80	58
	Reverse: TCAGGCTCTCCTGGTACTGGAT		
STAT5B	Forward: GGTCACGCAGGACACAGAGAA	110	58
	Reverse: CCAGCGGGCCAAACTG		
STAT6	Forward: CTTTTGGCAGTGGTTTGATGGT	96	60
	Reverse: TGTTTGCTGATGAAGCCAATG		
T-bet	Forward: AGGATTCCGGGAGAACTTTGA	123	60
	Reverse: TACTGGTTGGGTAGGAGAGGAGAGTA		
RORC	Forward: CCACAGAGACATCACCGAGCC	114	60
	Reverse: GTGGATCCCAGATGACTTGTCC		

### Statistical analysis

The results are shown as mean ± standard error of the mean (SEM). Data were plotted and analyzed using GraphPad Prism Software version 5.03. Student’s *t-*test was used for flow cytometry and real-time PCR analysis. One-way ANOVA with Dunnett’s test was used to determine the significance of difference between the controls and treatments. Two-way ANOVA with Bonferoni test was used to analyze statistical significance of the difference between means of cytotoxic experiments. A *p*-value < 0.05 was considered significant.

## Results

### The effects of AVS073 on the viability and proliferation of monocytes, PBLs and CIK cells

**Fig. 1:**
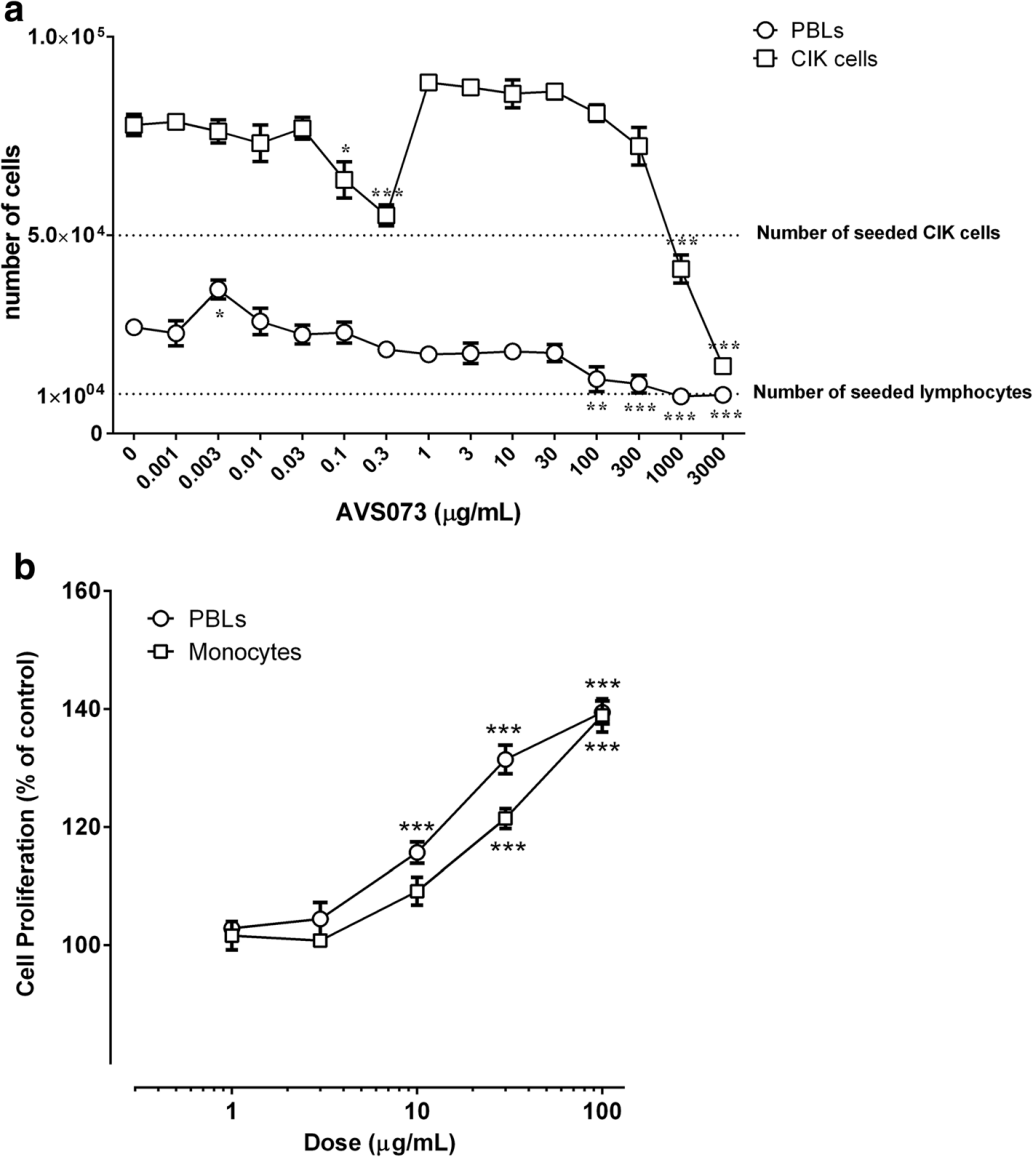
The concentration-toxicity relationship of AVS073 toward immune cells was evaluated using trypan blue assay and MTT assay to obtain suitable studying concentrations. The viability of PBLs (○) and CIK cells (□) after being exposed to AVS073 for 3 d was determined by trypan blue exclusion assay (**a**). Data are represented as mean ± SEM (*n* = 6). The MTT assay (**b**) demonstrated the proliferative effects of AVS073 on PBLs (○) and monocytes (□) after 7 d-culture. Data are represented as mean ± SEM (*n* = 8). *, ** and *** represent data with statistically significant difference from those of the untreated cells with *p* < 0.05, *p* < 0.01 and <0.001 respectively

AVS073 (1 ng/mL-3,000 μg/mL) were incubated with PBLs and CIK cells for 3d and determined for cell viability by trypan blue exclusion assay. The relatively low doses of AVS073 (1 ng/mL-30 μg/mL) did not harm the cultured cells. At higher doses (100–3,000 μg/mL), AVS073 compromised the viability of these cells (Fig. 1a). A range of 1–100 μg/mL AVS073 was brought to proliferation assay using MTT. The proliferation of these cells was highly improved after being cultured with 30–100 μg/mL of AVS073 (Fig. 1b). The viability of cultured cells after 3d was not compromised until the concentration reached 100 μg/mL. AVS073 at 30 μg/mL was selected for subsequent phenotypic and functional assays after being incubated with the selected immune cells for 7 d. The selected concentration did not compromise the mDCs’ viability (>90% viability) nor induce any alteration to cellular morphology and markers throughout the study (data not shown).

### The phenotypic characterization and the proportions of CD3^+^CD56^+^ and Treg subsets in PBLs and in CIK cells

**Fig. 2:**
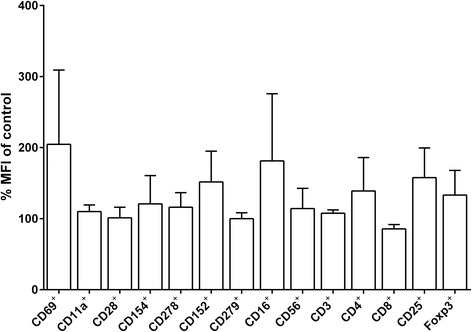
AVS073 was investigated for its potential action toward lymphocyte activation and differentiation in PBLs. The corresponding cellular markers were investigated using FACS. The mean fluorescence intensity (MFI) of lymphocyte markers was taken from FACS analysis of PBLs after the exposure to 30 μg/mL AVS073 for 7 d. Data are represented as mean ± SEM (*n* = 3) of % of untreated control. The unpaired *t*-test was used to analyze the statistically significant by compared with 100% control at *p* < 0.05

**Fig. 3:**
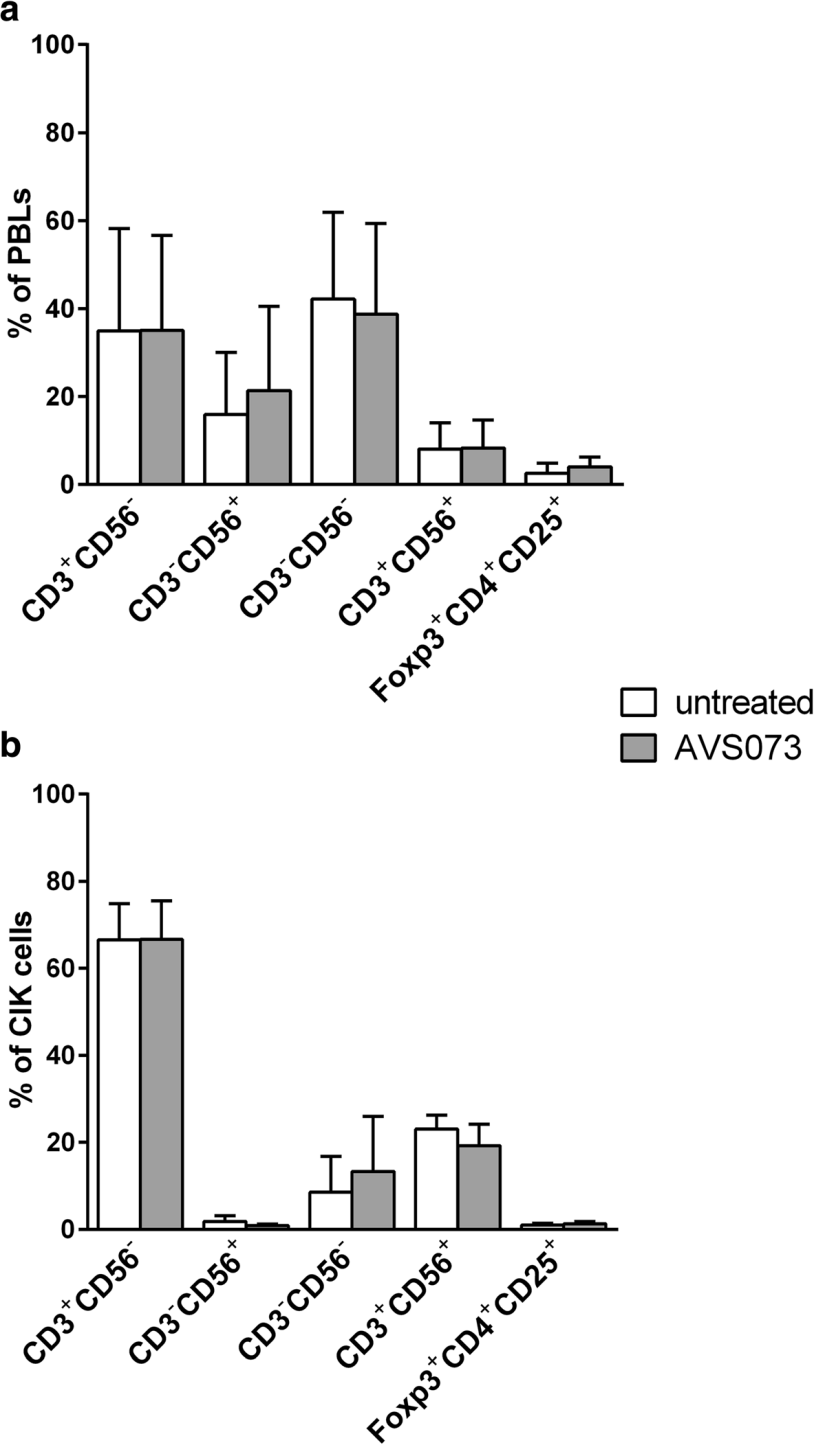
AVS073 was investigated for its potential action on the proportion of subsets in PBLs and CIK cells. The alteration in the proportions of CD3^+^CD56^−^, CD3^−^CD56^+^, CD3^+^CD56^+^ and Treg subsets was evaluated in PBLs (**a**) and CIK cells (**b**) after AVS073 exposure. Cells were cultured in presence or absence of 30 μg/mL AVS073 for 7 d. Data are presented as mean ± SEM (*n* = 3). The paired *t*-test was used to analyze the statistically significant at *p* < 0.05

There were no significantly alteration in the activation and differentiation markers (Fig. 2): including lymphocyte early activation marker (CD69), adhesion molecule (CD11a), cositmulatory molecules (CD8, CD28, CD154 and CD278), co-inhibitory molecule (CD152; CTLA-4) and negative regulatory molecule (CD279; PD-1), NK cell markers (CD16, CD56) and T cell markers (CD3, CD4, CD8, CD25, Foxp3) in PBLs after being treated with AVS073. Regarding the subset proportions, either PBLs (Fig. 3a) or CIK cells (Fig. 3b) were exposed to AVS073 for 7 d. There was no significant alteration in the proportions of CD3^+^CD56^+^, CD3^+^CD56^−^, CD3^−^CD56^+^, nor Treg subsets within both PBLs and CIK cells after AVS073 treatment.

### The analysis for the polarization of CD3^+^CD56^+^ cells after the exposure to AVS073

**Fig. 4:**
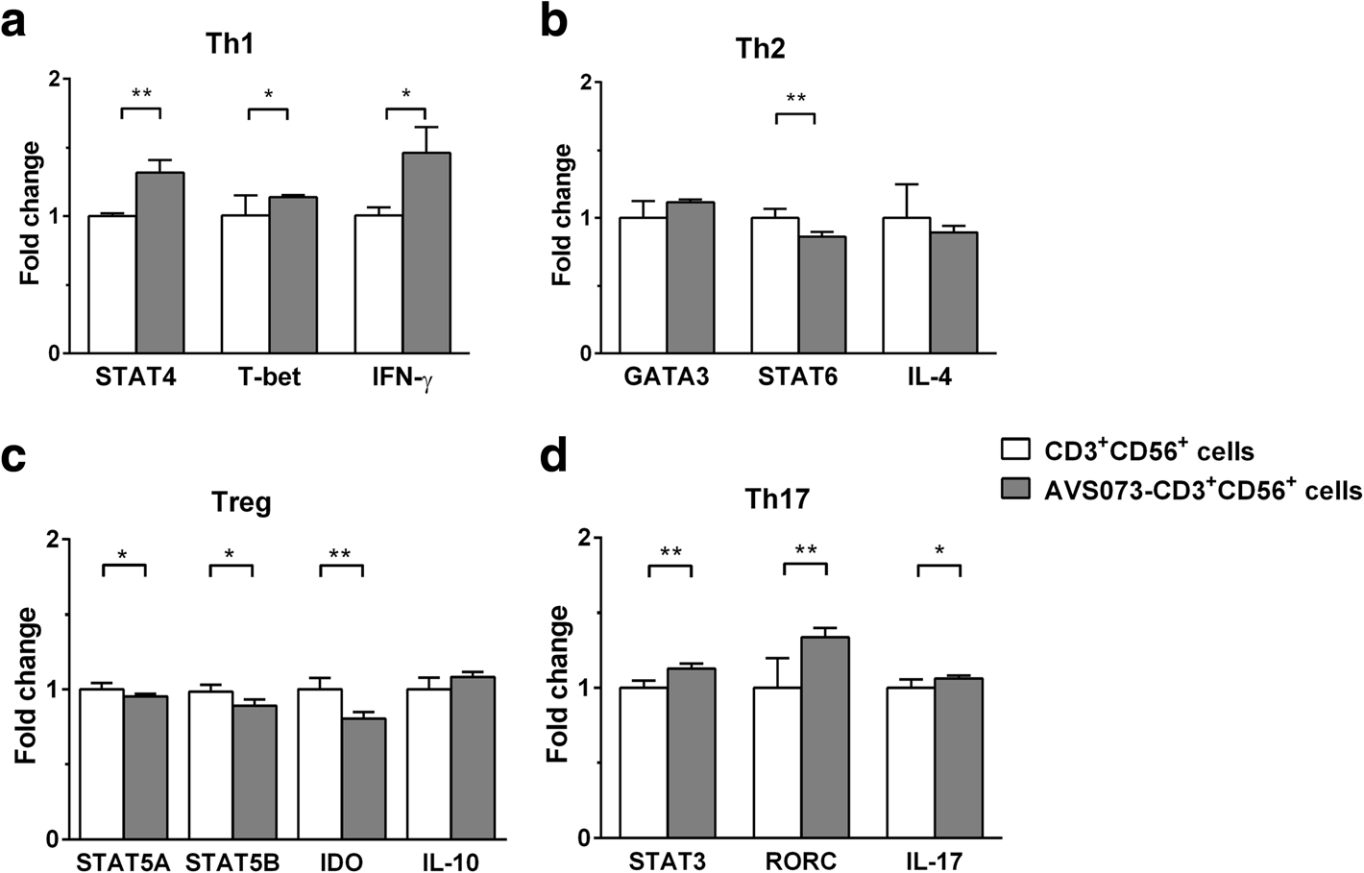
AVS073 was investigated for its potential actions toward the expression of polarization markers in CD3^+^CD56^+^ cells. The expression data of cytokines and transcription factors in CD3^+^CD56^+^ subset after the exposure to 30 μg/mL AVS073 for 7 d were evaluated using quantitative real-time PCR analysis. Data are represented as mean ± SEM (*n* = 4). * and ** represent data with statistically significant difference from those of the untreated cells with *p* < 0.05 and *p* < 0.01 respectively

The isolated CD3^+^CD56^+^ subset incubated with 30 μg/mL of AVS073 for 7 d were extracted for total mRNA and analyzed for the polarization markers. For Th1 markers, the expression of STAT4, T-bet, and IFN-γ, but not STAT1, were significantly increased (Fig. 4a). For Th2 markers, STAT6 expression was significantly decreased, but neither was GATA3 nor IL-4 (Fig. 4b). For Treg markers, STAT5A, STAT5B and IDO were significantly decreased, but neither was IL-10 (Fig. 4c). For Th17 markers, STAT3, RORC and IL-17 were increased (Fig. 4d).

### The phenotypic characterization of DCs after AVS073 treatment

**Fig. 5:**
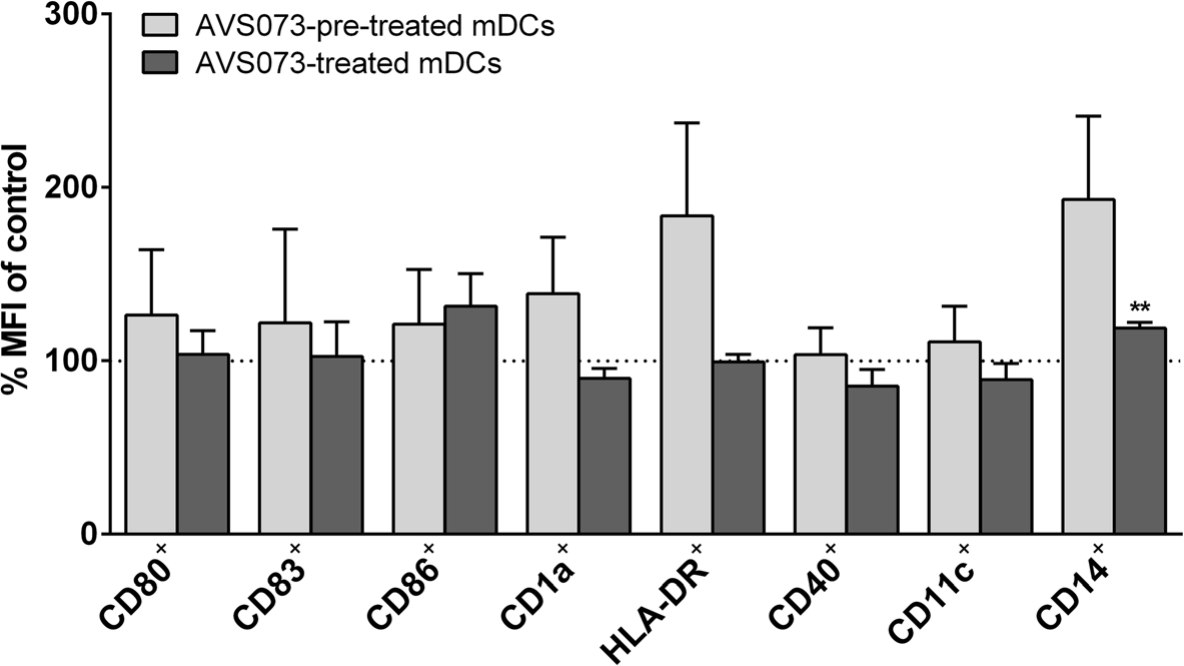
AVS073 was investigated for its potential action on the differentiation of mDCs. The MFI of differentiation markers on DCs after the exposure to 30 μg/mL AVS073 for 7 d were determined using FACS analysis. Data are represented as mean fluorescence intensity (% of the respective controls) ± SEM (*n* = 3). AVS073-pre-treated mDCs refers to DCs that exposed to AVS073 prior to TNF-α induced maturation. AVS073-treated mDCs refers to mDCs exposed to AVS073 after TNF-α induced maturation. The controls for AVS073-pre-treated-mDCs and AVS073-treated-mDCs were the untreated mDCs. * represents data with statistically significant difference from 100% control with *p* < 0.05

The expression of CD40, CD80, CD83 and CD86 were not altered in mDCs treated with AVS073 regardless of the treatment timing (Fig. 5). The expression of HLA-DR was not significantly altered in DCs regardless of the timing of AVS073 treatment. The expression of CD14, a monocyte marker, was suppressed in mDCs treated with AVS073.

### The alteration to the cytokines in mDCs after the exposure to AVS073

**Fig. 6:**
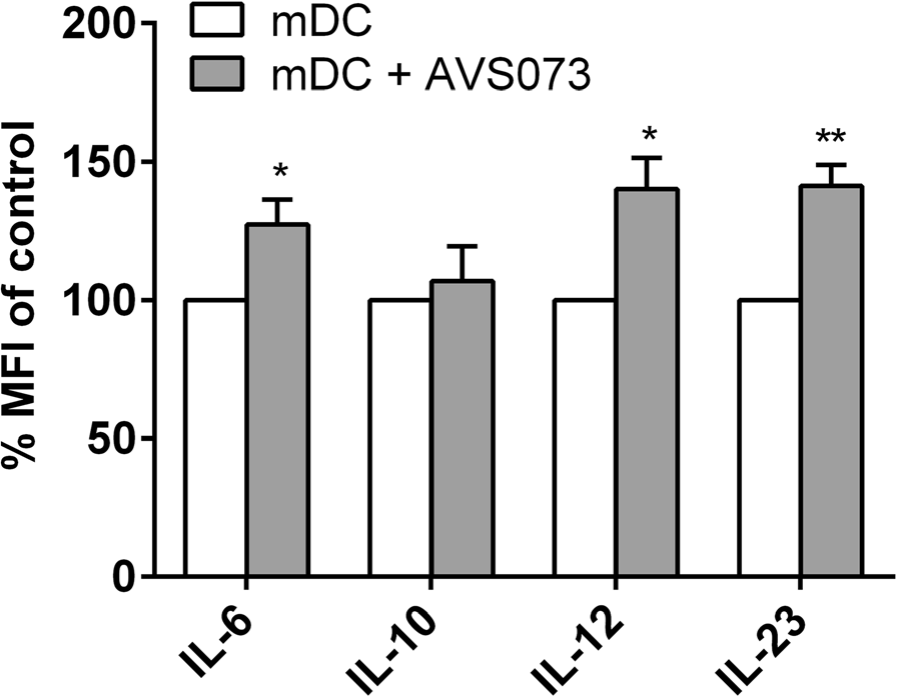
AVS073 was evaluated for its action on the polarization of mDCs. Flow cytometry analysis was used to evaluate the alteration of Th1/Th2/Th17 cytokines in mDCs after the exposure to 30 μg/mL AVS073 for 7 d. Data are represented as MFI (% of the respective controls) ± SEM (*n* = 6). The controls for AVS073-treated mDCs were the untreated mDCs. * and ** represent data with statistically significant difference from those of the untreated cells with *p* < 0.05 and *p* < 0.01 respectively

The level of IL-12, a Th1-prone cytokine, was rising in AVS073-treated mDCs (Fig. 6). AVS073 significantly increased the level of Th17-prone cytokines, IL-6 and IL-23 in AVS073-treated mDCs. The level of IL-10, an immunosuppressive cytokine that promote Treg development, was not altered.

## Discussion

### The phenotypic changes to PBLs

AVS073 did not alter the phenotypic characterization of PBLs: T-cell markers (CD3, CD4 and CD8), NK-cell markers (CD16 and CD56), activation markers (CD25, CD69), co-stimulatory molecules (CD28, CD40L, and ICOS), co-inhibitory molecule (CD152 or CTLA-4) or negative regulatory molecule (CD279 or PD-1). Moreover, AVS073 did not alter the proportion of Treg cells subset.

### The alteration to the CIK cell subsets

The absolute number of CD3^+^CD56^+^ subset at 21-d CIK cell increased up to 10 folds over that in the initial PBLs. AVS073 had not altered the proportion of CD3^+^CD56^+^ nor other subsets in both PBLs and CIK cells. The CD3^+^CD56^+^ subset was isolated from the CIK cells to monitor the alteration in Th1/Th2/Th17/Treg phenotypes after AVS073 treatment. The AVS073-treated CD3^+^CD56^+^ subset expressed more STAT4, that indicated the polarization toward Th1 phenotype. The Th2 and Treg phenotypes in AVS073-treated CD3^+^CD56^+^ subset was suppressed as evidenced by the decrease in STAT6 and STAT5A expressions, respectively. The Th17 phenotype was enhanced after AVS073 treatment as evidenced by increasing STAT3, RORC and IL-17 expression. Therefore, AVS073 tended to promote the Th1 and Th17 phenotypes in the CD3^+^CD56^+^ subset at the expense of Th2 and Treg phenotypes. The promotion of Th1 and Th17 phenotypes by the AVS073-treated CD3^+^CD56^+^ subset would make this preparation a candidate for anti-cancer treatment. Th17 cells exhibited pivotal roles in anti-tumor activity depending on the cytokines, costimulatory molecules and cell-cell interactions in the tumor microenvironment. The presence of IFN-γ together with IL-17 would promote tumor regression [53].

### Phenotype and maturation of DCs

The phenotypic changes induced by AVS073 might be attributed by its components. The *Carthamus tinctorius* L., one of AVS073′s components, was reported to promote the expression of maturation markers (CD80, MHC class I and II) in mouse bone-marrow-derived DCs and maintained the high profile of maturation markers (CD80, CD86, MHC class I and II) in tumor antigen pulsed-DCs [31]. In contrast, *Piper nigrum* Linn. extract, piperine, significantly inhibited the maturation (MHC class II, CD40 and CD86), cytokine production (TNF-α and IL-12), phosphorylation of ERK and JNK, but enhanced the endocytosis activity of LPS-induced bone-marrow-derived DCs [32]. However, we did not observe significant alteration in the maturation markers of both conditions of treated mDCs.

### Alteration to Th-polarizing profiles in DCs

The AVS073-treated mDCs carried more IL-12, the Th1-prone cytokine. AVS073 did not alter the IL-10 level, and therefore did not shift mDCs toward Treg-promoting activity. The cytokine levels of both IL-6 and IL-23 in AVS073-treated mDCs were increased, and therefore induced the Th17 polarization. IL-6 and TGF-β are necessary for the initial induction of Th17 differentiation whereas IL-23 is essential for the later stage of Th17 polarization [54]. IL-6 activates, while together with IL-21 further sustains STAT3 signaling required for ROR-α expression, and restrain Foxp3 mediated repression of ROR-α. TGF-β induces ROR-α expression through SMAD phosphorylation and provokes IL-23 receptor expression in naïve T cells, rendering them receptive to IL-23. IL-23 cements Th17 lineage commitment such that Th17 cells induced with TGF-β1 and IL-6 are insufficiently committed to the lineage and therefore plays a vital role in promoting Th17-mediated tissue inflammation [55].

The alteration to Th profiles would affect, in particular, immunocompromised patients. The Th1 and Th17 enhancing effects of AVS073 may boost protective adaptive immunity against infection and cancer while Th2-supppressing activity may also provide clinical benefit in allergic diseases including asthma. However, all mentioned have to be optimized with its effect on Treg suppression to prevent overt immune reaction. The pathogenesis of tumor immune evasion relies on immunosuppressive cells (e.g., Tregs) to establish an immunosuppressive tumor microenvironment. If this is the case, AVS073 can inhibit Tregs, and therefore attenuate tumor immune evasion and dampen cancer progression. Similarly, the pathogenesis of Th2-associated asthma is perpetuated by Th2 cytokine microenvironment that may be suppressed by AVS073 through downregulation of Th2 cells. However, we further need to study how to direct AVS073-treated CIK cells towards decisive immune boosting for reversing each disease condition as mentioned.

## Conclusions

The present study has demonstrated the effect of AVS073 on CIK and mDCs directing immune axis towards prominent Th1 and Th17 response while suppressing Th2 and Treg polarization. This molecular mechanism may provide clinical implication not only in boosting host protective immunity against infection and tumor immune evasion, but also reversing immune deviation in allergic diseases.

## Additional file

Additional file 1: The identity of mDCs was demonstrated using flow cytometry analysis for DC markers. The studied markers included CD80, CD83, CD86, CD40 and HLA-DR. (TIF 1013.76 kb)

## Abbreviations

AVS073: Ayurved Siriraj Wattana recipe
CIK: Cytokine-induced killer
DCs: Dendritic cells
FACS: Fluorescence-activated cell sorting
mDCs: Mature DCs
PBLs: Peripheral blood lymphocytes
PBMCs: Peripheral blood mononuclear cells
Treg: Regulatory T cell
