# Supplementary figures and images for: Effects of the Ayurved Siriraj Wattana recipe on functional and phenotypic characterization of cytokine-induced killer cells and dendritic cells in vitro
Source: BMC Complement Altern Med. 2016 Nov 29;16:489. doi: 10.1186/s12906-016-1480-7 (PMC5129228; doi:10.1186/s12906-016-1480-7)

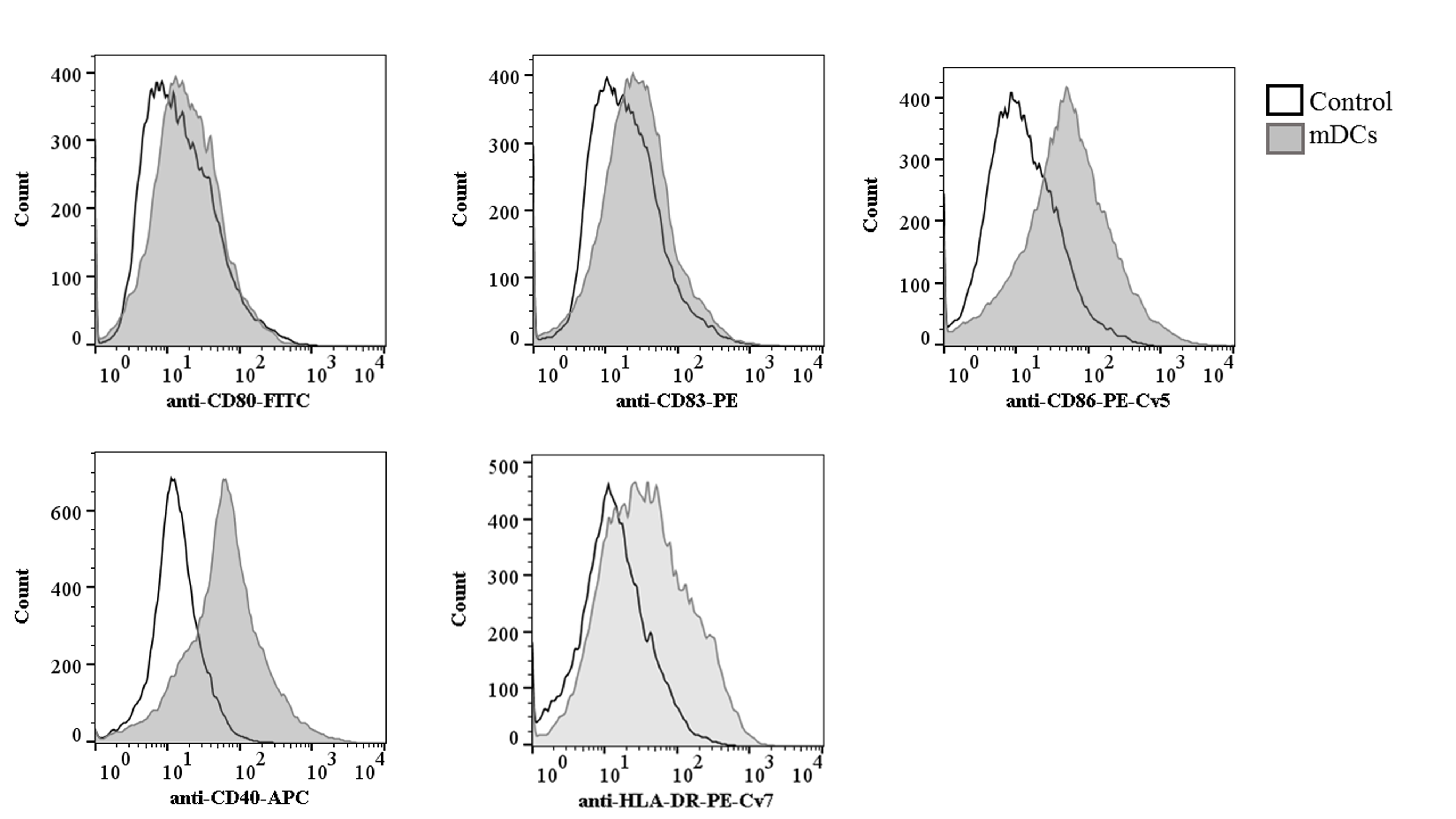

Supplement: Additional file 1: — The identity of mDCs was demonstrated using flow cytometry analysis for DC markers. The studied markers included CD80, CD83, CD86, CD40 and HLA-DR. (TIF 1013.76 kb) [file 12906_2016_1480_MOESM1_ESM.tif]
